# Antioxidant Activity of Co-Products from Guava, Mango and Barbados Cherry Produced in the Brazilian Northeast 

**DOI:** 10.3390/molecules19033110

**Published:** 2014-03-11

**Authors:** Kassandra L. G. V. Araújo, Marciane Magnani, Jaqueline A. Nascimento, Alline L. Souza, Poliana S. Epaminondas, Antônia L. Souza, Neide Queiroz, Antonio G. Souza

**Affiliations:** 1 Programa de Pós-Graduação em Ciência e Tecnologia de Alimentos, Centro de Tecnologia, Universidade Federal da Paraíba, Campus I, João Pessoa, Paraíba 58051-900, Brazil; 2 Laboratório de Bioquímica de Alimentos, Departamento de Engenharia de Alimentos, Universidade Federal da Paraíba, Campus I, João Pessoa, Paraíba 58051–900, Brazil; 3 Instituto Federal de Educação Ciência e Tecnologia, Campus Sousa, Paraíba, 58015-430, Brazil; 4 Departamento de Química, Universidade Federal da Paraíba, Campus I, João Pessoa, Paraíba 58051-900, Brazil

**Keywords:** juice processing, phenolic compounds, co-products, fruit pulp

## Abstract

Co-products from the juice processing of guava (CG), mango (CM) and barbados cherry (CB) were investigated with a view to their exploitation as a potential source of natural antioxidants. The ethanolic extracts were analyzed for total extractable phenolic content (TEP), DPPH radical scavenging activity (RSA-DPPH), ferric reducing antioxidant power (FRAP) and antioxidant activity in relation to the β-carotene/linoleic acid system. The TEP levels in the CG, CM and CB extracts were (24.15 ± 1.59), (44.18 ± 1.73) and (49.21 ± 3.70) mg GAE/g extract, respectively. The CM extract showed higher DPPH, FRAP and antioxidant activity in the β-carotene/linoleic acid system. The data revealed a positive linear correlation between TEP, RSA-DPPH and FRAP (r^2^ = 0.85 − 0.98); however, the β-carotene/linoleic acid system (r^2^ = 0.01 − 0.26) shows low correlation with the TEP levels and other assessment systems. The results suggest that co-products generated from the juice processing of the studied fruit have promising use as a natural source of antioxidants.

## 1. Introduction

Brazil is the sixth world’s largest producer of fruits, with an annual production exceeding 37 million tons, which represents 5% of global production [1]. Of the total production in Brazil, around 53% is aimed at the processed fruit market and 47% is fresh fruit [2]. In response to the high productivity of the processing sector and the favorable business results in the Brazilian fruit growing activity, there has been a considerable increase in fruit processing industries. However, these industries generate large amounts of co-products such as peels, seeds, and bagasse during fruit processing, which are usually inappropriately discarded in the environment, leading to waste accumulation and negative environmental impacts [3,4]. Like fruits, co-products contain antioxidant compounds, including polyphenols, carotenoids, ascorbic acid and tocopherols of great physiological importance [5]. In this sense, an effective option for adding value to these co-products is to extract any bioactive constituents that may be used in the pharmaceutical, food or cosmetics industries [3,6].

The number of studies using agroindustrial co-products as sources of antioxidants has increased considerably in recent years. Ajila *et al.* [7] have studied the bioactive compounds and the antioxidant potential of mango peel extracts using different measurement systems and reported high antioxidant activity. Due to this important property, these researchers suggested the use of mango peel as a nutraceutical functional food. Oliveira *et al.* [3] evaluated the antioxidant activity of methanolic extracts obtained from barbados cherry, passion fruit and pineapple industrial co-products and concluded that the barbados cherry flour has a high content of phenolic compounds and antioxidant activity against the DPPH radical, while passion fruit flour provides good protection against membrane lipid peroxidation. Babbar *et al.* [4] assessed the antioxidant activity and total phenolic compounds of powders from six co-products, and grape seed extract showed the highest content of phenolic compounds and highest antioxidant potential, and banana peel extract showed the lowest content of both. 

Several methods with different principles have been used in the *in vitro* antioxidant activity determination, among which the DPPH radical scavenging activity assay, which provides information on the overall antioxidant capacity of the test system [8] and the FRAP assay (ferric reducing antioxidant power) that relies on the ability of an anti-oxidant in reducing Fe(III) into Fe(II) and should be used combined with other methods because it cannot measure all antioxidants of complex matrices [9] stand out.. The β-carotene/linoleic acid coupled oxidation test is also used, where the model system is submitted to oxidation conditions that generate a free radical from the oxidation of linoleic acid, abstracting hydrogen from the unsaturated β-carotene molecule [10]. According to literature data, tests that evaluate antioxidant properties should employ more than one methodology in order to infer with greater certainty the antioxidant activity results [3].

Thus, the objective of this study was to quantify the total extractable phenolic compounds and to assess the *in vitro* antioxidant activity of co-products generated during industrial processing of guava *Psidium Guayaba* L.), mango (*Mangifera indica* L.) and barbados cherry (*Malpighia glabra* L.).

## 2. Results and Discussion

### 2.1. Yield and Total Extractable Phenolic Content (TEP)

The highest ethanolic extract yield of co-products from fruit processing was observed for the mango (CM; 11% ± 0.31%) and the lowest for the barbados cherry (CB; 5.5% ± 0.21%) (Table 1). However, the result found for CB was higher than that reported by Oliveira *et al.* [3] assessing methanolic extracts of barbados cherry (2.5%), pineapple (7.1%), and passion fruit (3.02%) co-products.

**Table 1:** Yield of extracts and total extractable phenolic content (TEP) of fruits co-products *.

Sample	Yield (%)	mg GAE/gof Extract	mg GAE/100 g of dc **
Guava	7.8 ± 0.29 ^b^	24.15 ± 1.59 ^b^	188.40 ± 12.38 ^c^
Mango	11.0 ± 0.31 ^a^	44.18 ± 1.73 ^a^	485.93 ± 19.08 ^a^
Barbados cherry	5.5 ± 0.21 ^c^	49.21 ± 3.70 ^a^	270.68 ± 20.37 ^b^

* Means (of the triplicate obtained in three different occasions) followed by different letters in the same column differs significantly (*p* ≤ 0.05); ** dc: dry co-products.

Phenolic compounds, present in large amounts in fruits, are reported as important constituents due to their antioxidant activity [11,12]. This power of scavenging radical structures results from the presence of at least one aromatic ring with hydroxyl groups on the chemical structure of phenolic compounds. The total extractable phenolic content (Table 1) ranged from 24.15 ± 1.59 to 49.21 ± 3.70 mg GAE/g of extracts and from 188.40 ± 12.38 to 485.93 ± 19.08 mg GAE/100 g in the dry co-products. The total extractable phenolic content in CB and CM extracts showed no significant difference between each other, reaching values two times higher than the extract CG. On the other hand, in dry fruit co-product , the TEP differed (*p* ˂ 0.05) and the highest values were observed in CM, followed by CB and CG. The results found in the CM extracts were lower than those reported by Kim *et al.* [13] for green and ripe mango peels (92.6 and 70.1 mg GAE/g each g of extract, respectively). However, like the results of this study, Ajila *et al.* [7] reported total extractable phenolic content values between 33.31 to 73.88 mg GAE/g of alcoholic extract, analyzing peel from two Indian varieties of green and ripe mangoes. The CB extract (49.21 ± 3.70 mg GAE/g) also showed differences in the total extractable phenolic content compared to the study by Oliveira *et al.* [3] (94.6 mg GAE/g) in methanolic extracts. However, Sousa *et al.* [2], observed total extractable phenolic contents from 46.77 to 279.99 mg GAE/100 g in wet co-product of fruit pulp extracted by a hydroethanolic process for guava and barbados cherry, respectively, which is equivalent to CB and lower to those reported for CG in this study. An interesting finding is that the value obtained for CG (188.40 ± 12.38 mg GAE/100g) was higher than that described for wet guava pulp (83.1 mg GAE/100 g) by Kuskoski, *et al.* [14], suggesting the potential use of co-products discarded during fruit processing. It is noteworthy that differences may also be due to factors such as climate, soil, stage of fruit ripening, drying method, type of solvent and extraction time, and part of the fruit that is present in the co-product [15].

### 2.2. Radical-Scavenging Activity of the 2,2 '-Diphenyl-β-Picrylhydrazyl Radical (DPPH)

Table 2 shows the antioxidant activity of each extract and BHT (used as reference), where it can be observed that the CM extract has the highest antioxidant activity among the analyzed extracts, followed by CB and CG extracts. The CM and CB extracts showed higher antioxidant activity (86% and 55%, respectively) when compared to BHT. Sousa *et al.* [2] have reported EC_50_ (µg/mL) of 142.89 and 308.07 for wet guava and barbados cherry co-products, respectively, and the value for barbados cherry is lower than that shown in this study. However, in dry products, the amount of phenolic compounds is concentrated.

**Table 2:** Antioxidant activity in ethanolic extracts of fruits co-products *.

Method	Guava	Mango	B. cherry	BHT
DPPH (EC _50_ µg/mL)	169 ± 6.00 ^a^	36 ± 0.58 ^c^	44 ± 4.36 ^c^	67 ± 3.06 ^b^
DPPH (anti-radical efficiency)	5.92 ± 0.21 ^d^	27.52 ± 0.43 ^a^	22.87 ± 2.15 ^b^	14.80 ± 0.66 ^c^
FRAP (mmol Fe(II)/g extract)	5.15 ± 0.57 ^c^	26.80 ± 0.63 ^a^	24.90 ± 0.17 ^b^	–
FRAP (mmol Fe(II)/100 g dc **)	40.15 ± 4.40 ^c^	273.86 ± 1.83 ^a^	147.44 ± 3.50 ^b^	–
β-Carotene bleaching (OI %)	60.64 ± 0.24 ^b^	88.91 ± 0.32 ^a^	39.36 ± 0.24 ^c^	–

* Means (of the triplicate obtained in three different occasions) followed by different letters in the same column differs significantly (*p* ≤ 0.05); ** dc: dry co-products.

**Figure 1:**
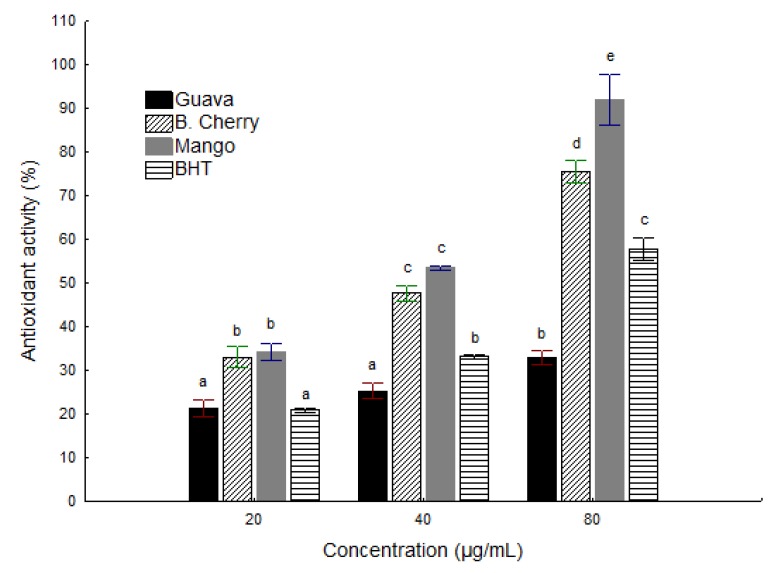
Antioxidant activity of extracts of fruit co-products compared with BHT against the DPPH radical.

Figure 1 shows that the ethanolic extracts of CB and CM have dose-dependent DPPH scavenging activity, differing from the highest concentration tested (80 mg/mL). The CG extract had an antioxidant activity equivalent to BHT at a concentration of 20 µg/mL, and lower at the other concentrations tested.

In a study by Babbar *et al.* [4] on methanolic extracts of lychee, tangerine, grapefruit and banana co-products at concentration of 5 mg/mL, it was observed that the co-products had antioxidant activity close to that of BHT at the same concentration (83%), except for banana peel, which was lower. Oliveira *et al.* [3] found antioxidant activity of approximately 82% for methanolic extract of barbados cherry powder co-product at concentration of 100 µg/mL, similar to results found here.

### 2.3. Ferric Reducing Antioxidant Power (FRAP)

Through the FRAP method, the CM extract also showed higher antioxidant activity, followed by CB and CG (Table 2). Muller *et al.* [16] reported FRAP values in barbados cherry of 17.23 mmol of Fe(II)/100 g of fresh fruit, and Rufino *et al.* [17] of 199.60 mMol Fe(II)/100 g of fruit on a dry basis. The value reported in this work for CB is higher than that found by these authors. This difference suggests that the content of antioxidant compounds present in seeds, the major component of co-products, is greater, which may contain compounds with higher antioxidant activity.

### 2.4. β-Carotene Bleaching Method

**Figure 2:**
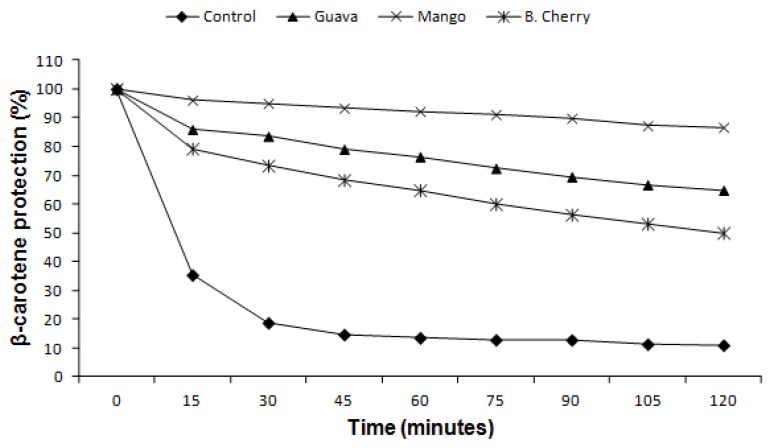
Protection of β-carotene oxidation added of extracts of fruits co-products.

All extracts at a concentration of 1 mg/mL inhibited β-carotene oxidation (Table 2). The CM extract, using both DPPH and FRAP methods, obtained the best result and the CG extract inhibited β-carotene oxidation more than the CB extract. The kinetics of β-carotene degradation (Figure 2) show that the extracts inhibit the β-carotene oxidation throughout the degradation curve both at the beginning of the oxidation process (15–45 min) as at the end of it (75–105 min). The CM extract gave better antioxidant activity when compared to CG and CB extracts. Importantly, in this system, barbados cherry co-products had a different behavior from that observed for FRAP and DPPH, which agrees with previous reports by Rufino *et al.* [17] and Alves *et al.* [18]. This change in behavior occurs due to the action of vitamin C, which acts as a pro-oxidant factor in the system with the formation of ascorbyl radicals during oxidation [17,19]. Another factor that may affect this result is the use of high temperature resulting in the decomposition of phenolic compounds [20].

### 2.5. Correlation between Total Phenolic Compounds and Antioxidant Activity using DPPH, FRAP and β-Carotene/Linoleic Acid Methods

The correlation between concentration of total phenolic compounds and antioxidant activity has been widely studied, including with fruits and fruit co-products [4]. In the present study, a high linear correlation (*r* > 0.92) was observed between the anti-radical efficiency in relation to DPPH and FRAP and total phenolic content present in the extracts tested, as reported by Oliveira *et al.* [3]. The antioxidant activity assessed by the β-carotene/linoleic acid system showed no linear correlation with the other methods tested or with the total phenolic content, as reported in the study by Rufino *et al.* [17]. It is known that not only phenolic compounds, but other components such as ascorbate, reducing carbohydrates, tocopherols, carotenoids, terpenes and pigments are factors that affect the antioxidant activity. In addition, each phenolic compound has distinct antioxidant potential, which in synergistic or antagonistic interactions, including with non-phenolic compounds, may affect the results [21].

## 3. Experimental

### 3.1. Reagents

The reagents 2,2-diphenyl-1-picrylhydrazyl (DPPH), 6-hydroxyl-2,5,7,8-tetramethylchoman-2-carboxylic acid (Trolox), 2,4,5-tris(2-pyridyl)-triazine (TPTZ), Tween^®^ 40, linoleic acid, butylated hydroxytoluene (BHT) were purchased from Sigma-Aldrich (Steinheim, Germany). Folin-Ciocalteau reagent and β-carotene were obtained from Merck (Dusseldorf, Germany). 3,4,5-Trihydroxy-benzoic acid (gallic acid), ferric choride hexahydrate, ferrous sulphate heptahydrate and other reagents and solvents used were of standard analytical grade.

### 3.2. Sample Preparation and Extraction

Co-products from the processing of guava (CG), mango (CM) and barbados cherry (CB) were obtained from a fruit pulp industry located in the city of João Pessoa/PB, Brazil. The fruit co-products collected directly from the production line were dried at 45 °C in an oven with air circulation (Model MA 035, Marconi, Piracicaba, Brazil) for 48 h. Then, the dried co-products were ground in a knife mill and vacuum packed in polyethylene bags and kept in a freezer (−18 °C) until the experiments. The extracts were obtained by stirring dry co-product (10 g) with ethanol (50 mL) in thermostated bath for 2 h at 25 °C. Then, the extract was centrifuged at 5,000 rpm for 10 min and the supernatant obtained was filtered with ethanol and diluted in a 100 mL flask. The extracts were stored in amber vials under an inert atmosphere until used. To determine the dry matter yield, 1 mL of extract was added to beakers previously dried in an oven and dried to constant weight at a temperature of 105 °C and the result expressed as percentage of extract/100 g of dry co-product (dc).

### 3.3. Determination of Total Extractable Phenolic Content

The levels of total extractable phenolic content (TEP) were determined colorimetrically by the Folin-Ciocalteau method [22] with volume reductions. Aliquots (4 mL) of ethanolic extracts diluted in water were added to Folin-Ciocalteau reagent (0.25 mL) followed by addition of 20% sodium carbonate (0.75 mL). The mixture was stirred and maintained in the dark for two hours. The absorbance at 765 nm was measured for UV-Vis spectrophotometer (model UV-2550, Shimadzu, Tokyo, Japan), together with a control containing only water and reagents. The concentration of phenolic compounds was estimated using a gallic acid curve calibration (50–500 mg/L). The results were expressed as mean ± standard deviation of mg gallic acid equivalent (GAE)/g of extract and GAE/100 g of dry co-product (dc).

### 3.4. Radical-Scavenging Activity of the 2,2'-Diphenyl-β-Picrylhydrazyl Radical (DPPH) Assay

The capacity of ethanolic extracts obtained from dry extracts to scavenge the DPPH radical was compared to the synthetic antioxidant BHT by the RSA-DPPH method proposed by Brand-Williams *et al.* [23]. Aliquots of ethanolic extract of each co-product and BHT (3.0 mL) at dilutions of 20, 40 and 80 µg/mL were added to DPP Hethanolic solution (0.1 mL, 0.1 mol/L). The control sample consisted each extract (3.0 mL) and ethanol (0.1 mL) and the negative control of ethanol (3.0 mL) and DPPH (0.1 mL). The decrease in absorbance at 515 nm was measured after 120 min. The RSA-DPPH was calculated according Rufino *et al.* [17]. From the results obtained, a graph for the % of DPPH radical scavenging activity in each extract concentration (mg/mL) was constructed. For the calculation of the EC_50_ (concentration of extract with the capacity to reduce 50% of the initial DPPH), the equation of a line was used, substituting the value of y by 50. The anti-radical efficiency was defined as the inverse relation of EC_50_, *i.e.*, (1/EC_50 (mg/mL)_).

### 3.5. Ferric Reducing Antioxidant Power (FRAP) Assay

The antioxidant activity of each sample was estimated by the ferric reducing antioxidant power (FRAP) assay [24]. Briefly, FRAP reagent (2.7 mL) prepared immediately before use (TPTZ 10 mM FeCl_3_and 20 mM acetate buffer) was homogenized with each extract (90 µL) and distilled water (270 µL) in a thermostated bath at 37 °C for 30 min. Then, the absorbance at 595 nm was measured using the FRAP reagent as negative control to calibrate the spectrophotometer. Concentrations of 500–2000 µmol/L of ferrous sulfate (FeSO_4_·7H_2_O) were used to determine the standard curve. The results were expressed as mean ± standard deviation of mM ferrous sulfate per g of co-product extracts of each fruit and mM ferrous sulfate per 100 g dry co-product (dc).

### 3.6. β-Carotene Bleaching Method

The antioxidant activity measured by the β-carotene/linoleic acid system model system co-product extracts was determined as method described by Miller [25]. This assay is based on the oxidation of β-caroteno/linoleic acid induced by oxidative degradation products of the linoleic acid. The solutions were prepared by mixing β-carotene/linoleic acid system solution (5 mL) and extract of each fruit (0.4 mL). After an initial absorbance reading at 470 nm, the mixture was kept in a thermostated bath at 40 °C, and then absorbance was measured at intervals of 15 min to 120 min. The negative control sample consisted of β-carotene/linoleic acid system (5 mL) and ethanol (0.4 mL). 

### 3.7. Statistical Analysis

All assays analysis was performed in triplicate in three different occasions. Results were expressed as mean ± standard deviation, compared by analysis of variance (ANOVA) and Tukey’s test, considering *p* < 0.05, using the Statistica 7.0 software (Statsoft^®^, Boston, MA, USA). The Pearson’s correlation coefficient (r) was calculated between total phenolic content and the methods used that asses the antioxidant activity.

## 4. Conclusions

Industrial co-products resulting from the processing of mango, barbados cherry and guava pulp are rich in total phenolic compounds, especially CM and CB. All ethanolic extracts tested showed effective antioxidant activity in the DPPH and FRAP assay systems and antioxidant activity in lipid systems, especially the CM extract. The results suggest that co-products from the processing of the three fruit pulps have promising use as sources of antioxidants.
